# The Effects of Particle Size Distribution and Moisture Variation on Mechanical Strength of Biopolymer-Treated Soil

**DOI:** 10.3390/polym15061549

**Published:** 2023-03-21

**Authors:** Hadi Fatehi, Dominic E. L. Ong, Jimmy Yu, Ilhan Chang

**Affiliations:** 1 School of Engineering and Built Environment, Griffith University, Nathan, QLD 4111, Australia; 2 Cities Research Institute, Griffith University, Nathan, QLD 4111, Australia; 3 Department of Civil System Engineering, Ajou University, Suwon-si 16499, Republic of Korea

**Keywords:** biopolymer-treated soil, biopolymer, durability under wetting and drying, agar, sodium alginate

## Abstract

Biopolymers have recently shown great potential to replace traditional binding materials in geotechnical engineering; however, more research is required to reach a deeper understanding of biopolymer-treated soil behavior. The objective of this study was to investigate the most important parameters that affect the behavior of biopolymer-treated soil, including biopolymer content, dehydration time, soil type effect, and durability. Sodium alginate and agar biopolymers were used due to their stability under severe conditions and the reasonable costs to study these parameters. A broad range of soil particle sizes was used to optimize the kaolinite-sand combination. As one of the main concerns in the behavior of biotreated soils, durability was investigated under five cycles of wetting and drying. In addition, a comprehensive microstructural study was performed by FTIR analysis and SEM images, as well as chemical interaction analysis. The results indicated that the optimized biopolymer content was in the range of 0.5–1% (to soil weight) and the dehydration time was 14 days. A soil combination of 25% kaolinite and 75% sand provided the highest compressive strength. Under wetting and drying conditions, biopolymers significantly increased soil resistance against strength reduction and soil mass loss. This study provides an understanding how agar and sodium alginate changes the behavior of the soil and can be used as a reference for further studies in the future.

## 1. Introduction

Soil treatment mainly focuses on the improvement of soil engineering properties such as strength, durability against wetting and drying cycles, and hydraulic conductivity. Biological techniques have recently been used in geotechnical engineering. Techniques such as microbial-induced carbonate precipitation (MICP) have proven to be effective in soil stabilization. The MICP requires a vast microbial community where cementation to the soil can provide a suitable condition for bacteria growth. [1,2,3,4,5,6,7,8].

Biopolymers are polymers that are naturally produced from different green sources, including fungus, algae or bacteria. They can be extracted from living organisms, synthesized chemically or derived from microbial systems [9]. Biopolymers increase the soil water retention capacity and, therefore, aid vegetation growth [10,11,12,13]. The compressive and shear strength properties of problematic soils have been significantly improved by adding various biopolymers, such as agar, xanthan, chitosan, alginate, and gellan gum [14,15,16,17,18,19,20,21,22,23,24,25]. 

Smitha et al. [26] conducted comprehensive research into the improvement of the mechanical properties and liquefaction potential of agar-treated silty sand. An increase in the agar content considerably enhanced the compressive and shear strength. Agar possesses hydrophobicity that excels in solubility and provides rigid textures when forming a gel [27]. Sodium alginate effectively improved the behavior of coarse and fine-grained soils in terms of shear strength and wind erosion resistance [17,28,29,30]. 

Biopolymer-treated soil is desired to have the ability to withstand climatic variations, especially when exposed to wet–dry cycles throughout seasonal changes [31]. Durability is related to the ability of soil particles and biopolymers to remain intact and hold together under wetting and drying. While there is potentially a significant variation in durability based on biopolymer type, limited research has been carried out to address this parameter. Chang et al. (2017) performed a durability study on treated soil using gellan gum as the binder. The performance of sand was improved after 10 cycles of wetting and drying by adding gellan gum [14]. Wind erosion and dust control of biopolymer-treated soil samples were evaluated after being subjected to wet–dry conditions. Biopolymers are able to increase the resistance of soil against wind erosion [32,33,34,35]. 

In view of the life cycle assessment for comparing the use of biopolymers and other conventional materials in a geotechnical project, biopolymers outperform conventional polymers considering the climate impact. Even though energy and water should be supplied during the synthesis and transportation of biopolymers, by using biopolymers the environmental impacts are reduced by approximately 85% compared with conventional polymers, and the impacts are significantly lower than those of traditional adhesives such as cement and lime [18]. In the case of agar, the conventional production process includes pre-treatment, extraction, filtration, concentration, and dehydration stages, which use very little water and leave a negligible carbon footprint. For alginate, its low toxicity, biocompatibility, and relatively low cost suggest that alginate is a good choice for ground improvement [18]. Alginate is produced in high amounts around the world with reliable sources [36]. Using hydrochloric acid in the production process of sodium alginate means that little energy is needed and low acidification, so sodium alginate yields a low environmental impact. Electricity accounts for 39% of the total impact and the use of chemicals accounts for 26%, on average [37].

This study aimed to evaluate the parameters that would impact the behavior of agar- and sodium alginate-treated soils, with a particular emphasis on soil particle size distribution and durability under cycles of wet–dry conditions. The study investigated the effects of different parameters on the characteristics of biopolymer-treated soils. Geotechnical and micro-structural tests, including unconfined compressive strength (UCS), scanning electron microscopy (SEM) images, and Fourier Transform Infrared (FTIR) spectroscopy analyses, were conducted. UCS tests were performed using different amounts of kaolinite and sand to investigate how biopolymer content, dehydration time, durability through wet–dry cycles, and soil particle size distribution change biopolymer strength. Additionally, to better understand the underlying strengthening mechanisms of the treated soils using biopolymers, SEM images, FTIR analysis, and a schematic soil and biopolymer interaction model were performed.

## 2. Materials and Methods

### 2.1. Sand and Kaolinite

**Figure 1:**
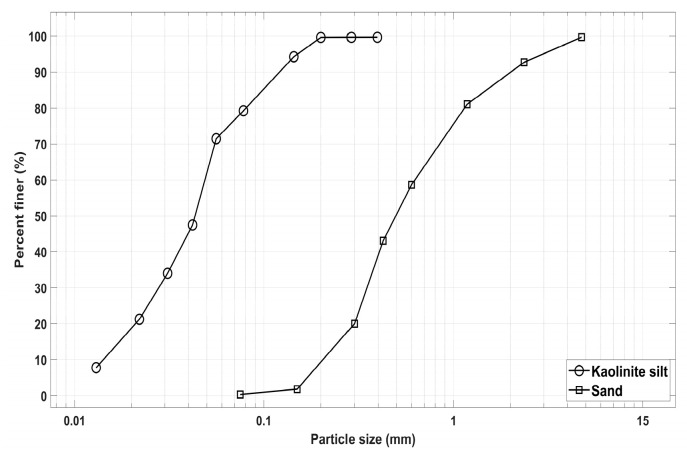
Gradation analysis of sand and kaolinite silt.

The sand used in the study was collected locally from a construction site located in Gold Coast City, Australia. The sand was classified as poorly-graded (SP) based on the Unified Soil Classification System (USCS). The gradation analysis of sand as well as kaolinite silt is demonstrated in Figure 1. Sand had a specific gravity of 2.63 and according to Figure 1, the uniformity coefficient (Cu) and the gradation coefficient (Cc) were 2.77 and 0.91, respectively. The basic geotechnical properties of the sand are shown in Table 1.

Kaolinite silt was purchased from Kaolin Malaysia, a company that provides kaolinite extracted from 4.0 to 6.0 m beneath the ground. The main chemical element of the kaolinite silt, according to the XRD study, is aluminum silicate. The geotechnical properties of kaolinite silt are shown in Table 1. The soil consists of silt (78%), clay (21.12%), and sand (0.88%) based on the particle size distribution and hydrometer analysis [38,39]. According to the USCS, the soil is classified as a high-plasticity kaolinite silt (MH). Table 2 shows the XRD analysis for sand and kaolinite used in this research. 

### 2.2. Agar Gum

Agar is a white to pale color, odorless biopolymer with the molecular formula C14H24O9. It is a strongly gelling hydrocolloid obtained from marine red algae. Agar is a heterogeneous mixture of two classes of polysaccharides: agaropectin and agarose. Agarose accounts for approximately 70% and agaropectin accounts for approximately 30% of agar. For this study, the agar gum was purchased from ChemSupply (Gillman, Australia) under the product name AGAR 1000 Gel (CAS 9002-18-0). Agar is negligibly soluble in cold water, while it is fully soluble in boiling water.

### 2.3. Sodium Alginate

Sodium alginate is used in the food and pharmaceutical industries for textile printing and paper coating, and in the cement industry as a thickener, stabilizer, and emulsifier. The sodium alginate used in this study was purchased from ChemSupply Australia (CAS No. 9005-38-3) (Gillman, Australia). The sodium alginate is a powder extracted from brown seaweed (alginic acid). Sodium alginate is soluble in water and turns into a paste at high concentrations. 

### 2.4. Sample Preparation

First, the soil was completely dried in an oven at a temperature of 105 °C. In order to mix the soil, water, and biopolymer, two mixing options were used—dry mixing and wet mixing. For dry mixing, biopolymer powder and soil were mixed before adding water. According to Fatehi et al. (2021), the wet mixing approach is the superior method, so it was used to achieve higher productivity [28]. For this purpose, the biopolymer and water solution was prepared. Due to the insolubility of agar gum in cold water, the water was heated to 100 °C, The biopolymer was then dissolved in the boiling water by mixing the two together for approximately 10 min until a homogenous solution was reached. For sodium alginate, the procedure was nearly the same; however, for sodium alginate, the water was heated to 70 °C. Then, the solution was added to the soil and mixed for 10–15 min. Finally, the biopolymer–soil mixture was maintained in twin sealed bags for 24 h to enable moisture to uniformly distribute throughout the mixture.

### 2.5. Kaolinite-Sand Combination

Sand-kaolinite combinations were mixed using kaolinite and dry sand at the mass ratios given in Table 3 to study the effect of soil type on the compressive strength of stabilized soil. For each kaolinite-sand combination, the variation in dry density with water content was demonstrated. In order to fabricate specimens using the optimum moisture content (OMC) and maximum dry density, the compaction tests were performed based on ASTM D698-12 [40]. The OMC obtained for K1S3 was 12.86%, as shown in Table 3, which was inadequate for preparing a homogenous biopolymer solution; therefore, for this case, water content was considered to be 15%.

### 2.6. Experimental Program

The compaction test, UCS test, unconsolidated-undrained triaxial test, FTIR, and SEM imaging were used to investigate the influence of biopolymers on soil engineering properties. The compaction test results were used to calculate the amount of water and soil required for each sample condition by measuring the maximum dry density and optimum moisture content. The UCS and triaxial tests were performed to evaluate the mechanical properties of the biopolymer-treated soil (BPTS), and FTIR spectroscopy and SEM images were conducted to identify the physiochemical characteristics of the treated soil. The experimental program of this study is shown in detail in Table 4.

A three-section labeling technique was used to distinguish between various specimens. The first section was used for the kaolinite–sand combination, which consisted of four parts: K stands for kaolinite (a number 0–4), S as sand, and a number (0–4). For example, K3S1 indicates a kaolinite-sand mixture consisting of three parts kaolinite and one part sand. The second section designates the biopolymers and their contents: SA for sodium alginate and Ag for Agar. The third is for indicating the dehydration days of the samples. For example, K2S2-SA0.5-14 refers to a soil with 50% kaolinite and 50% sand, which is stabilized by 0.5% of SA and cured for 14 days. For wet–dry cycle tests, because all durability samples were cured for 14 days, the last section is substituted with the number of cycles (from one to five).

#### 2.6.1. Unconfined Compression Strength Test

A unique mold was assembled to ensure the samples were compact and consistent in accordance with ASTM D4609 and ASTM D2850 [41,42]. The mold consisted of components including a mold cylinder (50 mm in diameter and 200 mm in height) with two welded wings on the side, a plug, and short and long hammers. The inside surface of the tube, hammer, and plug were moderately lubricated to facilitate the extrusion process. Then, the prepared biopolymer-soil mixture was gently poured into the mold in one layer. A hydraulic jack was employed to statically compress the sample to 95% of the maximum density obtained from the compaction test. The compressed sample was then extracted using the hydraulic jack and cured in a controlled room with a temperature of 23 °C and 50–60% relative humidity.

The compressive strength test was conducted using the Universal Testing Machine (UTM)—Instron 34TM-10 (Norwood, Massachusetts, United States) according to ASTM D2166 [41]. Factors such as biopolymer and moisture content, dehydration time, soil type effect, and durability of biopolymer-treated soil were examined using UCS tests. The axial strain rate was set at 1% per minute (1 mm/min) and continued up to 7% of the failure strain, with the machine automatically tracing and recording the stress-strain behavior. Three samples were tested to obtain a reliable average value for each condition.

**Figure A2:**
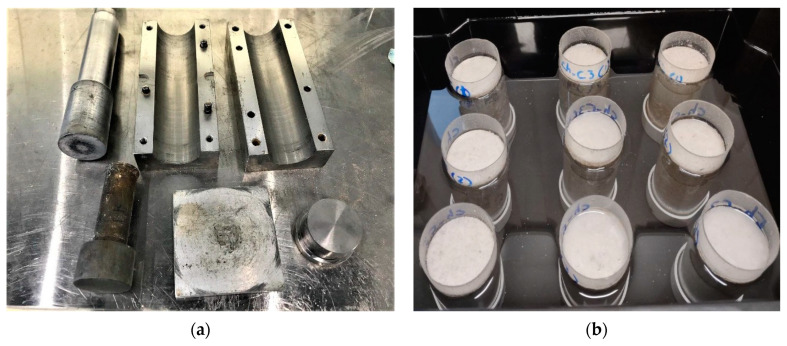
(**a**) UCS mold, (**b**) Wetting phase of wet–dry cycle.

Three specimens for untreated K1S3 and biopolymer-treated K1S3 samples were prepared for wet–dry conditions. Each sample was placed inside a plastic tube with a diameter of 55 mm, which was 5 mm larger than the sample diameter so the soil particles and biopolymer could move freely. The samples were soaked and kept for 24 h in a water-filled container. After 1 day of soaking, for the drying phase, samples were kept in the curing room for 14 days. The samples were put through a number of iterations (up to five) of drying and wetting and tested after cycles 0, 1, 2, 3, and 5. A picture of the UCS mold and the wetting phase of the wet–dry cycle can be found in Figure A2 in Appendix A.

#### 2.6.2. Attenuated Total Reflectance Fourier Transform Infrared (ATR-FTIR) Spectroscopy

Attenuated Total Reflectance Fourier Transform Infrared (ATR-FTIR) spectroscopy, abbreviated to ATR-FTIR, is a form of spectroscopy often used to study the chemical makeup of substances. To identify the functional groups of untreated and treated soil, ATR-FTIR analysis was performed using a Bruker Tensor27-spectrometer in the range of 400–4000 cm^−1^. A potassium bromide (KBr) disc technique in a ratio of 100:1 (KBr to soil) was used for sample preparation.

#### 2.6.3. Scanning Electron Microscopy (SEM) Images

After UCS tests, intact chunks of stabilized soil were subjected to SEM imaging to observe the microstructure of the samples. The samples were dried in the oven at 35 °C for 24 h. Then, they were attached onto a SEM mount by carbon conductive tabs. After ensuring sufficient electric grounding using carbon paint, samples were coated with a conductive tape. A Zeiss Sigma VP Field Emission Scanning Electron Microscope (Oberkochen, Germany) was used for specimen observation.

## 3. Results

### 3.1. Unconfined Compressive Strength Test 

The UCS tests were conducted to evaluate different parameters of the biopolymer-treated soil, including biopolymer content, dehydration time, soil type effect, and durability.

#### 3.1.1. Biopolymer Content

**Figure 2:**
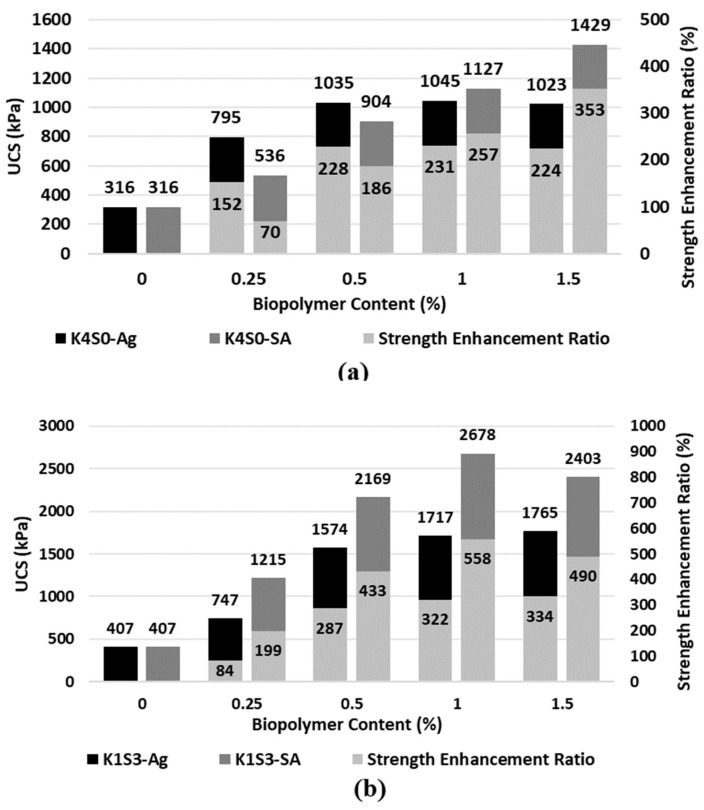

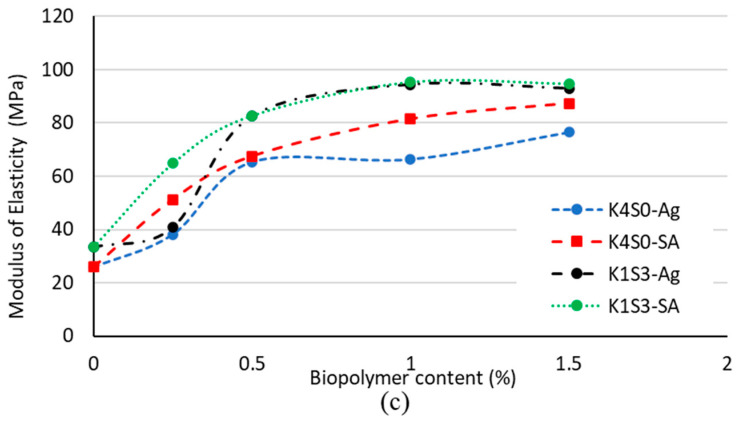
Biopolymer-induced Strength and enhancement ratio for treatment of (**a**) kaolinite, (**b**) K1S3, (**c**) modulus of elasticity.

Figure 2 shows the variation of UCS with biopolymer content for soil types of K4S0 and K1S3. Samples were cured for 14 days before the UCS tests. As obtained from the compaction tests, the initial moisture contents were taken as 35% and 15% for K4S0 and K1S3, respectively.

The UCS values of untreated soils were 316 and 406 kPa for K4S0 and K1S3, respectively, as shown on Figure 2. The results indicate a considerable improvement in compressive strength due to the addition of biopolymers up to 0.5%. This increasing trend remained promising for SA-treated samples up to 1%, while a small variation was observed for Ag-treated soils. For sodium alginate-treated K1S3, the compressive strength peaked at 2678 kPa (corresponding to 1% of biopolymer). This changing behavior is due to the formation of biopolymer gel higher than the amount required for binding soil particles. The lubrication effect of the extra gel spoils the binding interaction of the existing biopolymer gel and soil particles; therefore, the presence of extra gel results in less compressive strength due to the unconstrained movement of grains.

After comparing the results, the strength enhancement ratio values for the treated-K1S3 samples were found to be generally higher than the similar values of the treated-K4S0. For example, the increment rates for SA-treated K1S3 and K4S0 are 433% and 186%, and for Ag-treated K1S3 and K4S0 are 267% and 228%, respectively. This difference is in the soil particle size distribution, where a combination of fine and coarse-grained soils exists in K1S3, making it possible to achieve a higher density due to the fine particles of kaolinite that fill the pores among the sand grains. Additionally, reaching a uniform soil-biopolymer mixture was more difficult for K4S0 due to the light weight and hydrophobic carboxyl nature of kaolinite [43], while the sand in K1S3 facilitated the mixing procedure. 

Based on Figure 2c, the variation in the modulus of elasticity followed a similar trend to compressive strength, with the highest strength enhancement ratio being from 0 to 0.5% of biopolymer content. As expected, a greater modulus of elasticity values was recorded for the treated K1S3 samples compared to the treated K4S0. The cementation effect of adding biopolymers to sand and kaolinite enhanced both the compressive strength and modulus of elasticity. Various binding mechanisms are in place between biopolymers and the used soils including (a) hydrogen bonding between the hydroxyl surface of kaolinite and functional groups of the utilized biopolymers, (b) electrostatic interaction between the charged surfaces of kaolinite and biopolymers, and (c) hydrophobic bonding. Moreover, the distribution of polymeric chains of biopolymers throughout the soil void space and coating the sand surfaces create a strong and integrated film network across the treated soil mass, which improves the resistance forces and prevents the movement of soil grains.

#### 3.1.2. Dehydration Time

The dehydration time and moisture content effect on the compressive strength of agar and sodium alginate-treated soils (K4S0 and K1S3) were investigated. For this purpose, 0.5% of biopolymer was used and samples were tested after 1, 3, 7, 14 and 28 days of dehydration in a controlled room (23 °C in temperature and 60 % humidity). Three samples were prepared for each condition and the average was reported.

**Figure 3:**
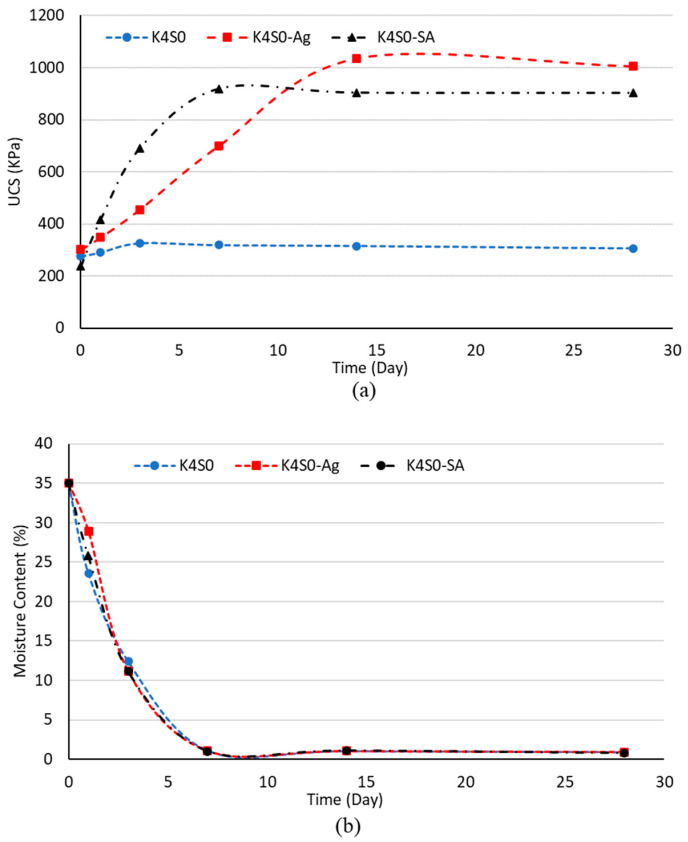
Effect of dehydration time on biopolymer-treated K4S0 (**a**) UCS, (**b**) moisture level.

Figure 3 shows the UCS variation in terms of dehydration time, obtained by the UCS tests for treated and untreated kaolinite soil. Untreated and treated samples yielded almost similar compressive strengths immediately after specimen fabrication (0 days of curing), suggesting the ineffectiveness of biopolymer gel in the very early stages of dehydration. Over time, the compressive strength of the samples increased. In the case of sodium alginate-treated kaolinite, more than 90% of the compressive strength was achieved within the first 7 days of dehydration, and strength remained nearly constant until day 28 after peaking on the day 7. On the other hand, for Ag-treated kaolinite, the maximum strength was reached during the first 14 days of dehydration.

The moisture content has a considerable influence on the rheological and mechanical characteristics of the biopolymer gel: the greater the moisture level, the lower the molecular weight and viscosity. Therefore, the stiffness of the polymer increases over time (as the moisture content decreases) and lowers its deformability significantly, resulting in increased compressive strength in the treated soil samples. The results are approximately similar for treated and untreated samples, and the moisture content decreases over time until 7 days of dehydration, where it starts to remain approximately constant. In the first days of dehydration (0 to 3 days), treated samples contained more moisture compared to their untreated counterparts, which can be related to the water absorbed by biopolymers.

**Figure 4:**
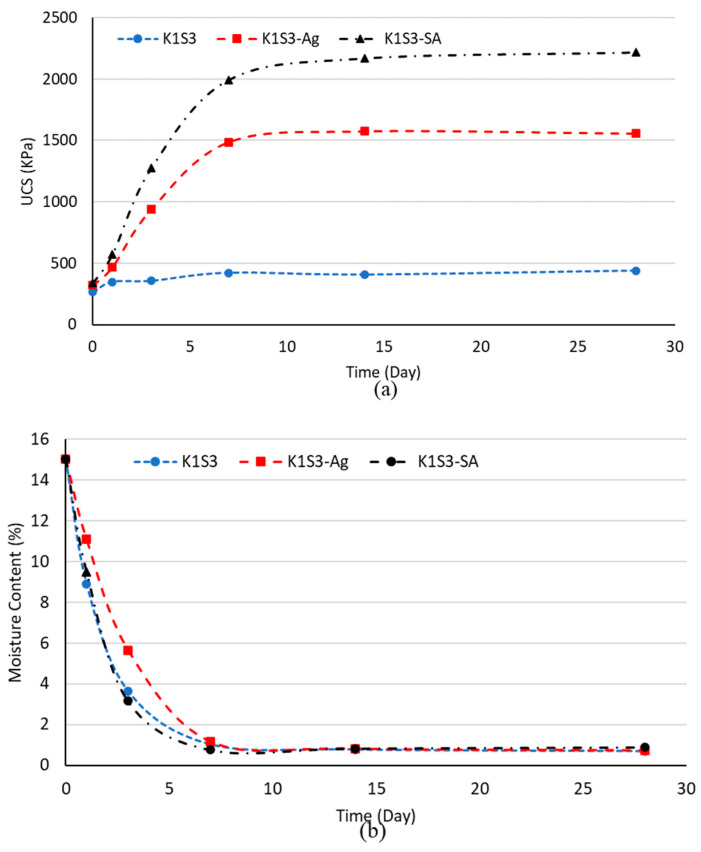
Biopolymer-treated K1S3 variation over time (**a**) UCS, (**b**) moisture level.

Figure 4 shows the comparable strength values and moisture contents obtained for agar and sodium alginate-treated K1S3 soil over time. The results are almost similar to that of kaolinite. As seen, the strength of the treated specimens substantially increased until day 7. Samples achieved maximum strength within 7 days until day 14 of dehydration and remained almost constant until day 28. As a result, in subsequent stages of the study, 14 days was chosen as the optimum dehydration time.

#### 3.1.3. Soil Type

**Figure 5:**
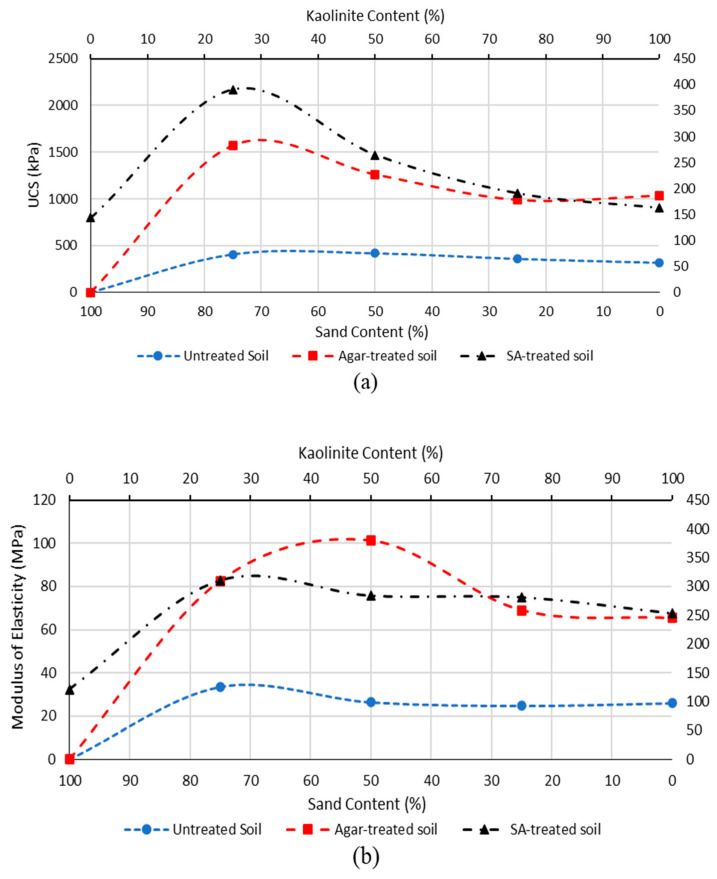
Soil type effect on characteristics of treated soil (**a**) UCS, (**b**) modulus of elasticity.

The influence of different soil types (treated and untreated) on the UCS and modulus of elasticity was investigated by conducting UCS tests. Stabilized samples were treated with 0.5% of sodium alginate and agar biopolymers and cured for 14 days. The results are represented in Figure 5. The moisture content of the samples was less than 1%, so moisture effect was neglected. 

K1S3 was the best soil sample in terms of compressive strength, showing the highest compressive strength in both treated and untreated states, as shown in Figure 5. The high UCS can be attributed to the size distribution (better graded) of the soil K1S3, because the kaolinite particles fill the pores between sand particles. Therefore, the solid mass of K1S3 can be denser due to the interlocking of the particles, which enables it to support heavier loads. When the samples were treated with 0.5% of biopolymers, they demonstrated a considerable enhancement in UCS compared to untreated samples. Treated K1S3 showed the highest UCS strength enhancement ratio, meaning that biopolymers were the most effective for K1S3, while biopolymer-treated river sand showed the lowest UCS strength enhancement ratio, indicating the soil type least influenced by biopolymers. Generally, sodium alginate performed better than agar and led to increased compressive strength in the samples. Moreover, Figure 5b shows the modulus of elasticity obtained for various soil types: modulus of elasticity shows approximately the same behavior as UCS.

#### 3.1.4. Wet–Dry Cycles

**Figure 6:**
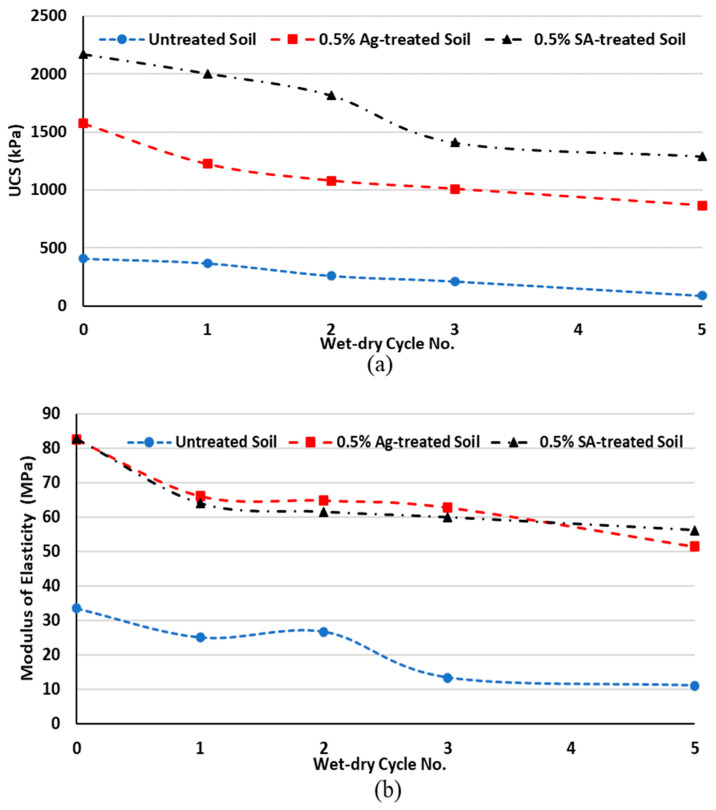
Effect of wet–dry cycles on the (**a**) UCS and (**b**) modulus of elasticity of biopolymer-treated K1S3.

The durability of treated (with 0.5% of biopolymer) and untreated soil was examined using UCS tests after wet–dry cycles. To this end, K1S3 was chosen as the soil type. Figure 6 represents the UCS and modulus of elasticity of K1S3 samples (cured for 14 days) after 1, 2, 3 and 5 wet–dry cycles. It can be observed that wetting and drying significantly reduced the strength and modulus of elasticity of the samples. Untreated samples lost approximately 78% of their initial strength following five cycles of wetting and drying, while treated samples performed better. Sodium alginate-treated K1S3 had a better performance compared to its agar-treated counterparts. SA-treated K1S3 lost 40% of its initial strength after five wet–dry cycles, whereas Ag-treated K1S3 lost 45% of its initial strength after the same number of wet–dry cycles. Permeating water to the soil pores increases the pore pressure, which results in strength reduction and soil loss in the untreated soil. However, for biopolymer treated soil, while soaking in water reduces strength, part of the bonding and interactions are regained during the drying process for BPTS; therefore, a smaller number of soil grains are able to escape from the soil mass. In terms of stiffness, biopolymer-treated samples experienced a modest drop in modulus of elasticity, which is consistent with the UCS findings. Therefore, alginate and agar reduce the likelihood of soil strain softening.

**Figure 7:**
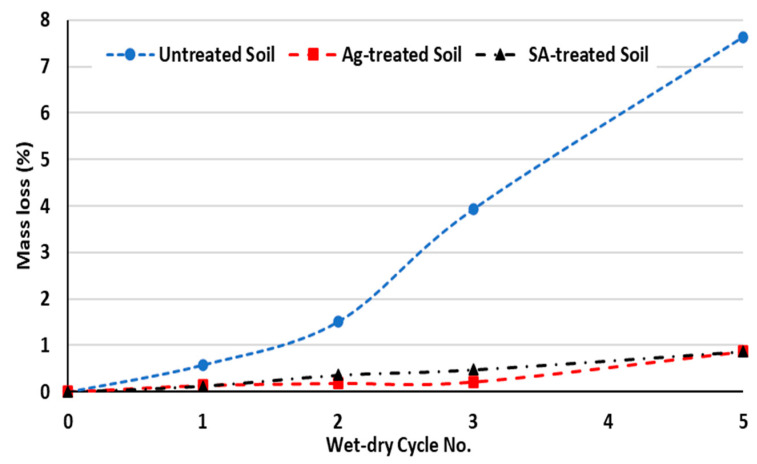
Mass loss of soil specimens with repetitive wet–dry cycles.

**Figure 8:**
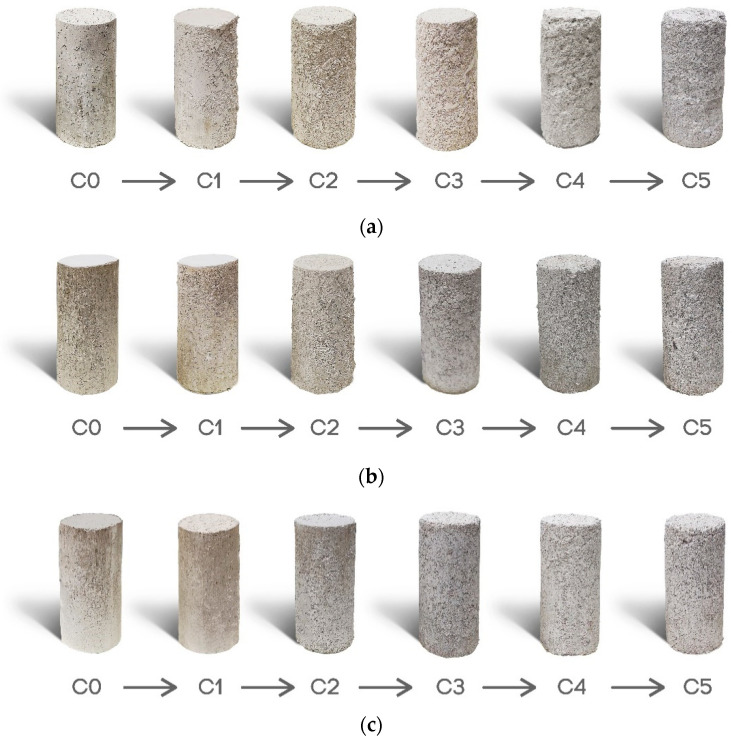
Treated and untreated soil samples from cycle 1 to cycle 5. (**a**) untreated, (**b**) agar-treated, and (**c**) sodium alginate-treated samples.

The mass loss of the samples under durability testing is shown in Figure 7 and Figure 8. As expected, the mass loss related to untreated samples is more significant than that of treated samples after passing through five wet–dry cycles. For example, after five wet–dry cycles, untreated samples lost approximately 8% of their mass, while the mass loss for treated samples was less than 1%. 

Because of the mass reduction, the resulting cross section may be decreased which reduces the stress to particles and can result in reduced strength. Additionally, biopolymer films are exposed to water during wetting, which can cause the detachment of some hydrated gel fibrils from the main structure and soil particles. The detached film reattaches to the main structure during the subsequent dehydration cycle due to moisture loss and a small percentage of the original structure may not be recovered after each cycle. The side effects of this cyclic degradation are less modulus of elasticity of the gel, a decrease in gel density, and a higher maximum strain [14]. This explains why treated soils experience a progressive loss of strength during wet–dry cycles rather than a rapid failure, as can be seen with lime- and cement-treated soils [44,45,46].

### 3.2. Microstructure and Interaction Model

**Figure 9:**
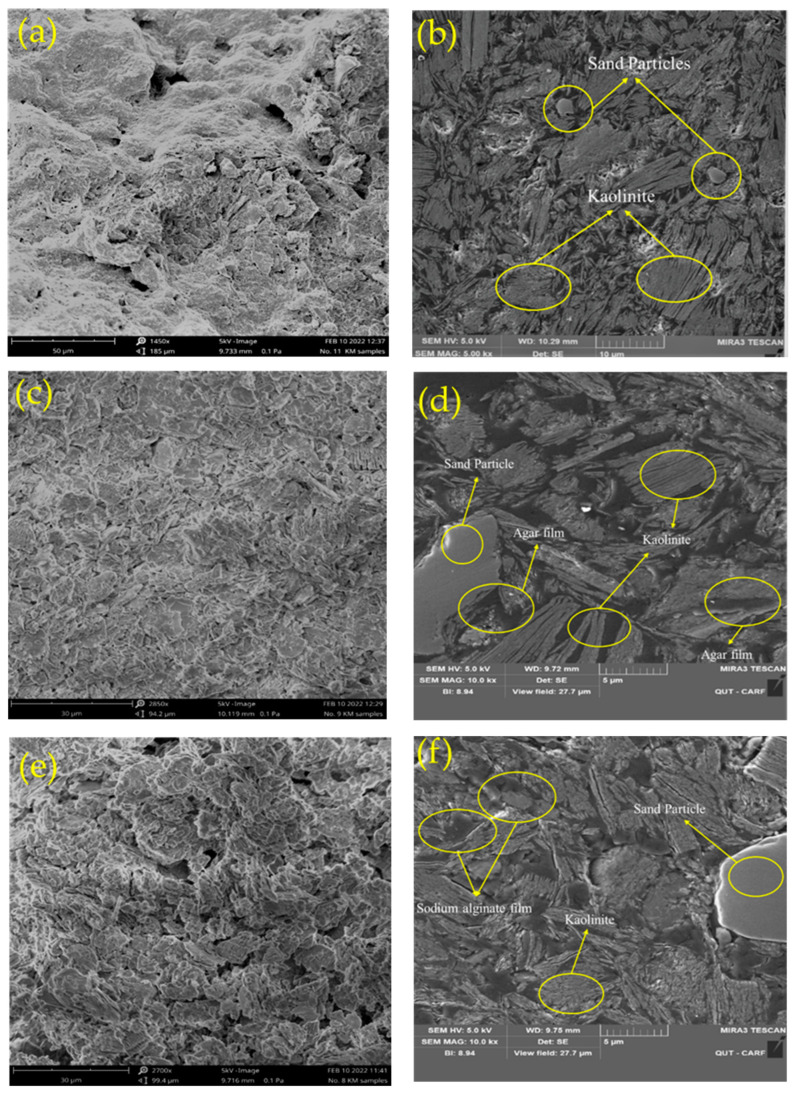
SEM images of (**a**) Kaolinite, (**b**) K1S3, (**c**) K4S0-Ag, (**d**) K1S3-Ag, (**e**) K4S0-SA, (**f**) K1S3-SA.

This section explains the possible interactions that occur during the creation of kaolinite/biopolymers and sand/biopolymers composites. SEM photos of the untreated and biopolymer-treated samples are presented in Figure 9. Detailed explanations for the mechanism of each soil-biopolymer combination are provided in the following sections.

#### 3.2.1. Kaolinite and Sodium Alginate Composite (Kaolinite/SA Composite)

**Figure 10:**
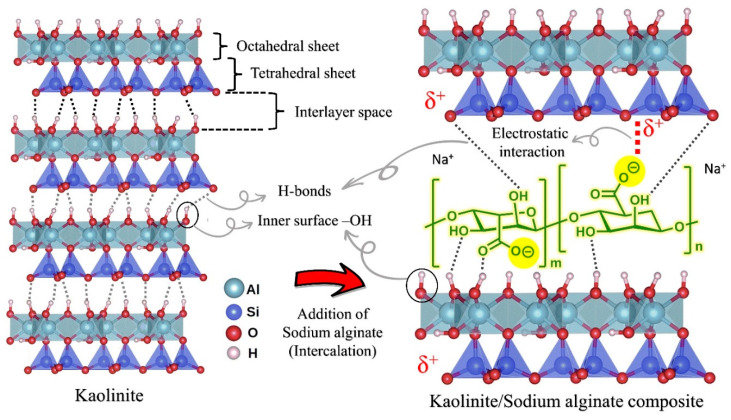
Molecular structure of sodium alginate, kaolinite and their interactions.

The molecular structure of sodium alginate and kaolinite is illustrated in Figure 10. Sodium alginate is a charged polymer containing carboxylate and hydroxyl functional groups. Kaolinite possesses surface hydroxyl (alominol, Al–OH) and oxygen groups (Si-O-Si), which can establish effective hydrogen bonding with organic polymers bearing electronegative functional groups [47]. In examining the possible interactions between sodium alginate polymer and kaolinite particles in their composite structure, three major interactions can be found:

(a) Hydrogen bonding: effective hydrogen bonds can be formed between the surface hydroxyl groups of kaolinite (alominols) and the ketone (in the carboxylate group) and the hydroxyl functional groups in the sodium alginate structure (either by intercalating or entering the polymer chain of sodium alginate within the kaolinite plates or by hydrogen bonding between SA and the outer surface of the kaolinite).
(b) Electrostatic attraction: carboxylate functional groups bear negative charge (–COO–) onto the structure of the sodium alginate chain, which can have an effective electrostatic interaction with the positive surface charge of the kaolinite layer, as shown in Figure 10.
(c) Hydrophobic bonding: hydrophobic bonding between the carbon chain of the sodium alginate polymer and the outer surface of the kaolinite layers without functional groups (uncharged basal surface of kaolinite) can also be established in the kaolinite/sodium alginate composite structure.

#### 3.2.2. Kaolinite and Agar Composite (Kaolinite/Agar Composite)

**Figure 11:**
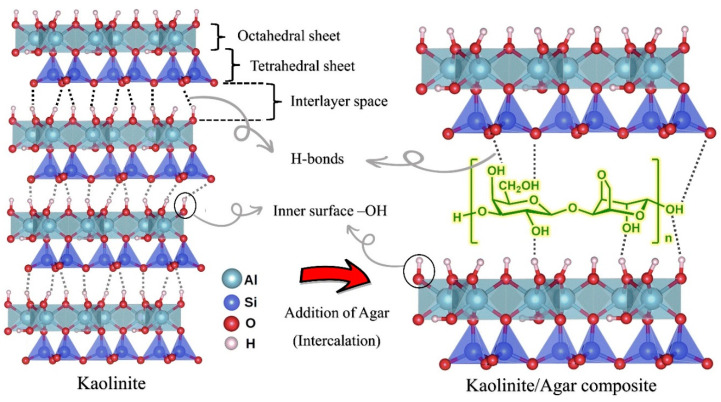
Structure of kaolinite and agar and their interaction.

Agar is a non-ionic linear polymer consisting of a mixture of two polysaccharides: agarose (70% of the mixture) and agaropectin. Agarobiose (a disaccharide made up of D-galactose and 3,6-anhydro-L-galactopyranose) is a repeating unit of agar as shown in Figure 11 [48]. The hydroxyl group (–OH) is the only active functional group in the structure of the agar that can establish effective hydrogen bonding with materials bearing hydrogen attached to electronegative groups as well as electronegative elements such as oxygen. This means that hydrogen bonding between agar and a substance can occur either through an electrostatic-type interaction (called H-bonding) between the hydrogen of a hydroxyl group of agar and an electronegative atom with lone-pair electrons (such as oxygen), or through an electrostatic-type interaction between a lone-pair electrons of a hydroxyl group of agar and hydrogen atoms covalently attached to the electronegative atoms of every substance [49]. Regarding the possible interactions involved in the formation of a kaolinite/agar composite, two main interactions can be proposed: (I) H-bonding interaction between hydroxyl groups of agar and surface hydroxyl groups (alomino, Al–OH) of kaolinite (either by intercalating and entering the polymer chains of agar between the kaolinite plates, or by hydrogen bonding with the outer surface of the kaolinite), (II) common intermolecular or interparticle interactions called hydrophobic bonding, which occurs between the outer surface of the kaolinite layers (uncharged basal surface of kaolinite) and the uncharged carbon chain of agar.

#### 3.2.3. River Sand and Agar/Sodium Alginate Composite

**Figure 12:**
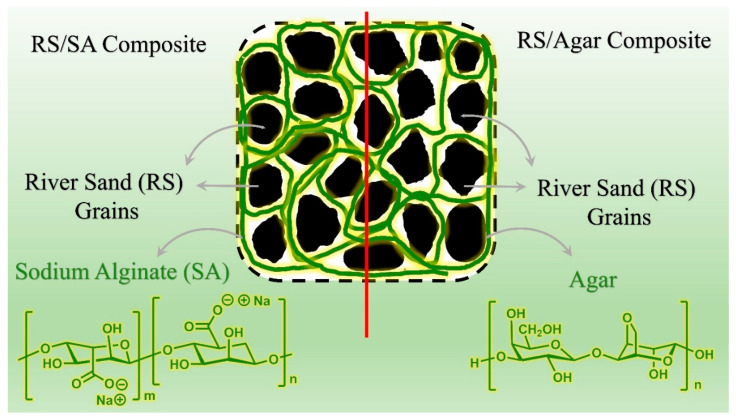
Structure of river sand, sodium alginate and agar and their interaction.

As shown in Figure 12, considering the uncharged structure of river sand (RS) particles as well as the nonpolar polymer structure of agar, the possible dominant interactions in their composite formation can be demonstrated as the following [50]:

(a) Hydrophobic bonding: since agar is an organic linear polymer without an electric charge and RS grains are assumed to be uncharged, hydrophobic interaction between them can be assumed when they come in contact on a microscopic surface. This is the predominant interaction in such compounds with a same nonpolar of uncharged nature.
(b) Interfacial force of RS/agar fibers: the interfacial mechanical interaction between organic fibers of agar and particles of RS can lead to the formation of a homogeneous composite structure. The retaining mechanical force created by the placement of polymer fibers around the RS grains is a positive driving force that improves the mechanical strength of the grains in the composite structure compared to the pure structure of the RS in the absence of fibers.

### 3.3. ATR-FTIR Spectra of K1S3-SA and K1S3-Ag

**Figure 13:**
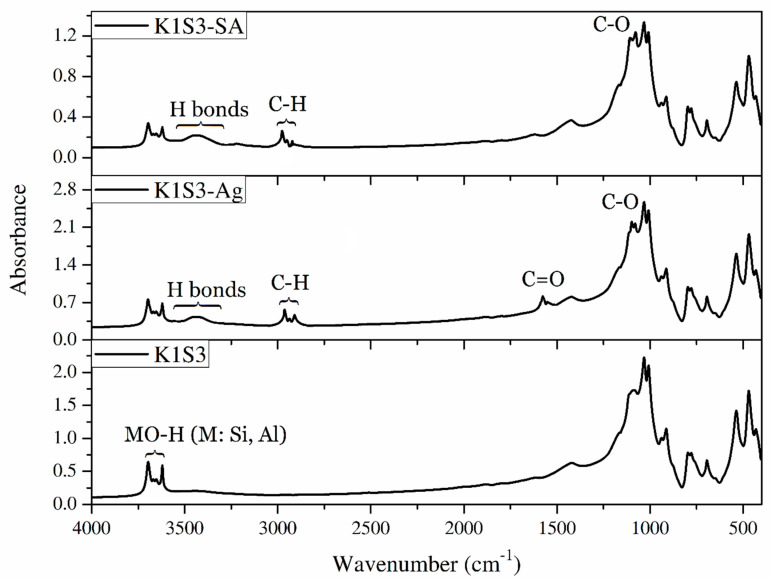
FTIR analysis of treated K1S3 using agar and sodium alginate.

As shown in Figure 13, the ATR-FTIR spectrum of K1S3 represents the characteristic absorption signals related to kaolinite: Si–O–Al group (at around 795, 700, and 534 cm^−1^), Al–OH group (at 911), Si–O group (at around 1080, 1030, and 1007 cm^−1^), and –OH group (at 3694, 3664, 3648, 3621 cm^−1^). In the case of –OH absorption bands between 3694 and 3620 cm^−1^, the signals attributed to the outer –OH appeared at 3621 cm^−1^, and the signals related to the inner –OH were observed at 3694, 3664, and 3648 cm^−1^ [51]. In comparison with K1S3, the ATR-FTIR spectrum of both composites shows absorption signals from approximately 1170 to 930 cm^−1^ (overlapped, C–O stretching), 2866 cm^−1^ (C–H symmetric stretching), and 2908 cm^−1^ (C–H asymmetric stretching), which is representative of functional groups of organic moieties in the composite structures. Moreover, for K1S3-Ag, the presence of ATR-FTIR signals at 1615 cm–1 is attributed to the carboxylate anions (C=O stretching vibration). Additionally, relatively broad hydrogen bond signals caused by hydroxyl (in both agar and sodium alginate) and carboxylate anions (in agar) groups, as well as surface adsorbed water, could be observed in the spectra of composites. These aforementioned characteristic signals are representative of the presence of organic moieties (Ag and SA) in the composite structures and, accordingly, the successful preparation of K1S3-SA and K1S3-Ag composites.

## 4. Conclusions

The feasibility of employing two popular biopolymers, sodium alginate and agar, was investigated in this study. This study investigates the key factors affecting the mechanical behavior of biopolymer-treated soils and the interactions of soil and biopolymers. A conclusion of the attained results is as follows:

Both biopolymers considerably improved the compressive strength of soil. More than 85% of the maximum strength was obtained by adding 0.5% biopolymer to the weight of soil. Therefore, the optimal additive content to reach the highest strength was 0.5%.

Moisture content decreased over time, resulting in an increase in the compressive strength. For different soil types, the optimum dehydration time can be considered to be 14 days since no significant improvement was observed between 14 and 28 days.

Soil with clay-to-sand particle sizes was tested in order to obtain the optimized combination. Based on the obtained results, a soil mixture comprising 75% sand and 25% kaolinite performed best in terms of compressive strength.

After being subjected to five wetting and drying cycles, the strength loss of biopolymer-treated soils was reduced from 78% for untreated soil to less than 40% for biopolymer-treated soil, respectively. Similarly, mass loss of the soil decreased from 8% to less than 1%, respectively.

## Figures and Tables

**Table 1:** Geotechnical properties of the used soils.

**Sand**	D_50_ (mm)	C_u_	C_c_	G_s_	Shape	UCSC	e_min_	e_max_
	0.49	2.77	0.91	2.63	Round	SP	0.61	0.76
**Kaolinite**	Sand fraction (%)	Silt fraction (%)	Clay fraction (%)	L_L_ (%)	P_L_ (%)	P_I_ (%)	USCS	Activity = P_I_/Clay content (%)
	0.88	78.00	21.12	62	46	16	MH	0.77

**Table 2:** XRD analysis of the used soil in this study.

Sand		Kaolinite Silt	
Chemical Analysis	Characteristic (%)	Chemical Analysis	Characteristic (%)
Quartz	48.97	Silica	48.97
Calcite	35.19	Aluminum	35.19
Dolomite	2.51	Potassium	2.51
Siderite	0.88	Iron	0.88
Siderite (Mg/Ca)	0.35	Manganese	0.35
Andradite	0.23	Titanium	0.23
Plagioclase	7.7	Calcium	0.03
K-Feldspar	3.0	Phosphorus	0.05
Illite	3.2	Manganese	0.1
Chlorite	1.4	Others	**11.5**

**Table 3:** Sand-kaolinite mixtures.

Label	Soil (%)			Optimum Moisture Content (%)	Maximum Dry Density (gr/cm^3^)
	Sand	Silt	Clay		
K4S0	0	80	20	35.13	1.37
K3S1	25	60	15	29.88	1.57
K2S2	50	40	10	20.25	1.75
K1S3	75	20	5	12.86	2.04
K0S4	100	0	0	16.75	1.83

**Table 4:** Experimental program of the current study.

Test Type	Biopolymer Type	Biopolymer Content (%)	Dehydration Time (Days)	Soil Type	No. of Wet–Dry Cycles
UCS	SA, Ag	0.25, 0.5, 1, 1.5, 2	14	K4S0, K1S3	-
	SA, Ag	0, 0.5	0, 1, 3, 7, 14, 28	K4S0, K1S3	-
	SA, Ag	0, 0.5	14	K4S0, K3S1, K2S2, K1S3, K0S4	-
	SA, Ag	0, 0.5	14	K1S3	0, 1, 2, 3, 5
FTIR	SA, Ag	0.5	14	K1S3	-
SEM	SA, Ag	0.5	14	K4S0, K1S3, K0S4	-

**Figure A1:**
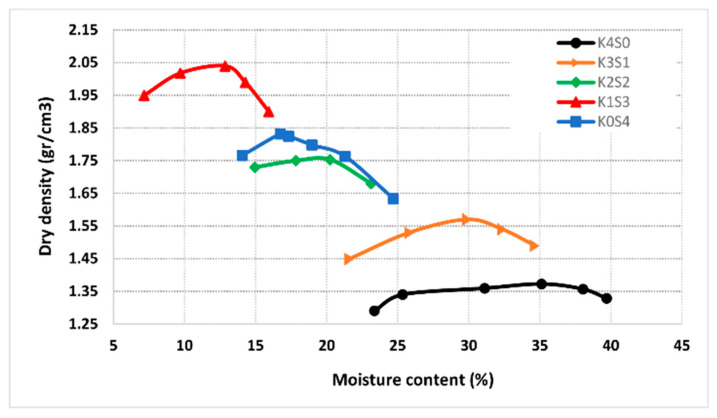
Compaction curves for sand-kaolinite mixtures.

## Data Availability

There is not data to be publicly shared.

## Appendix A

Supporting information of the study has been provided in Appendix A.
